# The clinical applicability of percutaneous splenic vein stent implantation for pancreatic portal hypertension

**DOI:** 10.1186/s12876-022-02214-z

**Published:** 2022-03-25

**Authors:** Jingjing Liu, Qingbing Wang, Xiaoyi Ding, Qin Liu, Wei Huang, Junwei Gu, Zhongmin Wang, Wei Wu, Zhiyuan Wu

**Affiliations:** 1 Department of Interventional Radiology, Ruijin Hospital, Shanghai Jiao Tong University School of Medicine, Shanghai, 200025 China; 2 Department of Gastroenterology, Ruijin Hospital, Shanghai Jiao Tong University School of Medicine, Shanghai, 200025 China

**Keywords:** Hypertension, Portal, Splenic vein, Pancreatitis, Chronic, Self expandable metallic stents, Splenectomy

## Abstract

**Background:**

Pancreatic portal hypertension (PPH) is a type of extrahepatic portal hypertension. We compared the clinical efficacy of different treatment methods for PPH caused by splenic vein stenosis in chronic pancreatitis.

**Methods:**

This article retrospectively analyzed the PPH cases that were caused by splenic vein stenosis after chronic pancreatitis. Patients were divided into three groups according to the different treatments: splenic vein stent implantation (stent group), splenectomy, and only medications (conservative group). The treatment effects from each group were compared.

**Results:**

A total of 33 patients were retrospectively analyzed in this study (9, 12, and 12 patients in each group respectively). All the procedures were successful in the stent and splenectomy groups. During the follow-up, no patient had gastrointestinal bleeding recurrence in the stent and splenectomy groups. However, in the conservative group, the incidence of portal hypertensive gastropathy and upper gastrointestinal bleeding were 50% and 25%. In the stent group, all the varicose veins at the base of the stomach had shrunk by varying degrees, and the red color signs regressed. The stent patency rate was 100%. No major complication occurred. The average platelet count at 1, 3, 6-months postoperatively were all significantly higher than the preoperative value (P < 0.05). The average postoperative hospital stay duration was significantly shorter than that of the splenectomy group (3.1 ± 1.4 days vs. 16.1 ± 8.1 days; P < 0.05). In the splenectomy group, postoperative fever occurred in 4 patients. Postoperative infection occurred in 2 patients (one with abdominal cavity infection and the other with incision infection). Delayed abdominal bleeding occurred in one patient. Portal vein thrombosis occurred in 2 patients during follow up.

**Conclusion:**

Percutaneous splenic vein stent implantation for PPH treatment reduces the risk of gastrointestinal bleeding with minimal invasive. It has a high safety and reliable efficacy and is worthy of further clinical promotion.

**Supplementary Information:**

The online version contains supplementary material available at 10.1186/s12876-022-02214-z.

## Background

Pancreatic portal hypertension (PPH), also known as pancreatic regional portal hypertension, is a type of extrahepatic portal hypertension. It is caused by obstruction of the portal vein system branches or by obstruction of the venous return after pancreatic disease or related complications [1]. To date, it is the only type of portal hypertension that can be cured [2]. Although it only has an incidence of approximately 5% in all portal hypertension patients [3], in this condition, gastric and/or esophageal varices are common and can cause upper gastrointestinal bleeding, which can be life-threatening [4]. At present, the main PPH treatments methods include splenectomy, endoscopic sclerotherapy, endoscopic ligation, etc. [5]. PPH is often due to local splenic vein stenosis or occlusion. If the splenic vein can be recanalized, then the portal hypertension might be relieved effectively. In this study, through retrospective analysis, we compared 3 different PPH treatment methods: percutaneous splenic vein stenting, splenectomy, and conservative treatment only with medications.

## Methods

### Clinical data

We retrospectively analyzed 33 patients with PPH that were admitted to our hospital between April 2015 and October 2021. The patients’ general informations were showed in Table 1. Patients were divided into the following groups according to the different treatments: splenic vein stent implantation (stent group), splenectomy, and only medications (conservative group). This retrospective study was performed in accordance with medical ethics regulations.

**Table 1:** General information

	Stent group	Splenectomy group	Conservative group
Number of cases	9	12	12
Male:female ratio	8:1	10:2	11:1
Average age	46.22 ± 9.42	49.58 ± 10.80	38.08 ± 7.40
*Clinical manifestations*			
Abdominal pain	3	9	11
Gastrointestinal bleeding	6	4	0
*Preoperative laboratory indicators*			
WBC < 3 × 10^9^/L	5	3	1
Hb < 90 g/L	4	3	0
PLT < 100 × 10^9^/L	7	3	3
*Preoperative portal CTA*			
Splenic vein occlusion	4	4	3
Splenic vein stenosis	5	8	9
*Preoperative EUS*			
Gastric varicose veins	9	12	12

WBC, white blood cell count; Hb, hemoglobin; PLT, platelet count; CTA, computed tomography angiography; EUS, endoscopic ultrasound

*Inclusion criteria:* All patients met the diagnostic criteria for PPH [6] and met the following inclusion criteria: (1) chronic pancreatitis; (2) clinical, endoscopic, or laboratory evidence proving that there was portal hypertension with complete or partial splenic vein occlusion.

*Exclusion criteria:* (1) Portal hypertension with a non-pancreatic cause, such as liver cirrhosis;(2) PPH with pancreatic tumors (including benign and malignant); (3)other malignant tumors; (4) serious impairment of important organs (such as the heart, lung, kidney, brain, etc.) or of blood coagulation.

Indications for splenic vein stents or splenectomy: patients with chronic pancreatitis who had the history of gastrointestinal bleeding due to regional portal hypertension or at higher risk of bleeding as evidenced by gastroscopy. In patients with splenic vein stent implantation, intraoperative splenography were also required to confirm severe splenic vein stenosis (> 70%).

### Treatment approach of each group

**Fig. 1:**
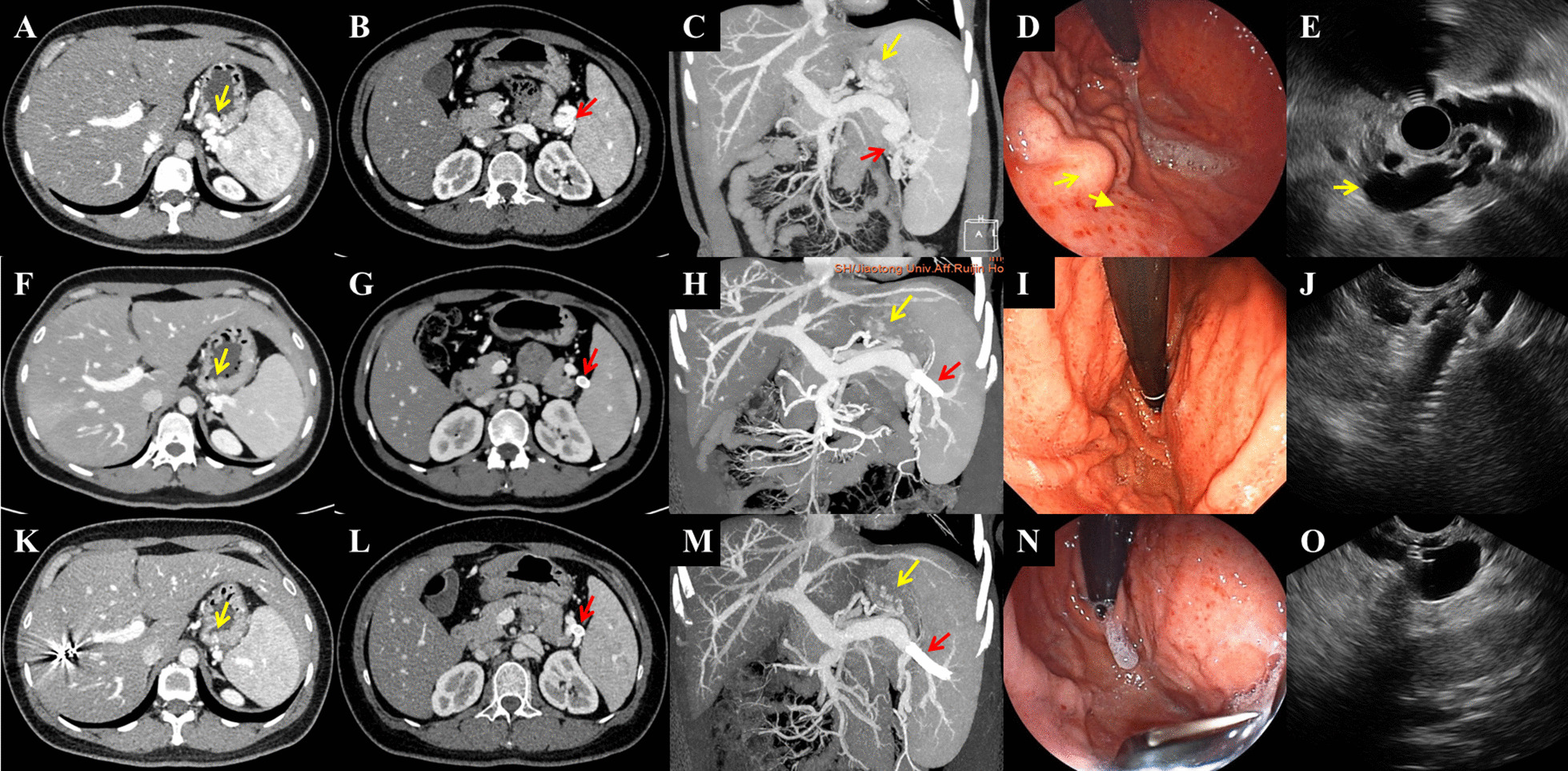
Preoperative and postoperative computed tomography findings from the patients in the stent group. Computed tomography (CT) scan and endoscopy of a 36-year-old woman before and after stent insertion. **A**–**C** The preoperative contrast-enhanced CT showed severe splenic vein stenosis (red arrow), obvious gastric varices (yellow arrow), and an enlarged spleen. **D** The preoperative upper GI endoscopy showed marked gastric varices (yellow arrow) in the fundus and along the greater curvature of upper gastric corpus. **E** Radial EUS showed enlarged vascular lumen of the splenic vein around the hilus of spleen (yellow arrow), with blocked vein drainage and multiple submucosal fundal varices ranging from 5 to 7 mm in diameter. **F**–**H** Three months post-operatively, when the patient was re-examined, the contrast-enhanced CT scan showed that the main splenic vein and its branches were unobstructed. The stent was in place (red arrow) and the varicose veins at the base of the stomach (yellow arrow) had shrunk significantly. **I** Endoscopy performed 3 months post-operatively revealed significant regression of gastric varices. **J** Linear EUS showed tip of stent in the lumen of remnant splenic vein near the hilus of spleen. **K**–**O** Thirteen months post-operatively, the contrast-enhanced CT scan and endoscopy showed no recurrence of varicose veins at the base of the stomach (yellow arrow)

Computed tomography (CT) is an excellent tool to assess the collateral patterns and to determine the underlying cause [7]. So all the patients underwent evaluation of the pancreas, liver, and portal vein system through contrast-enhanced abdominal CT (Fig. 1). Gastroscopy and/or ultrasound gastroscopy were performed to evaluate the gastroesophageal varices (Fig. 1).

Complete routine blood, coagulation, liver and kidney function, and amylase tests were performed. Patients in the stent and splenectomy groups fasted for 8 h before the operation and signed the preoperative consent form.

### Stent group treatment approach

**Fig. 2:**
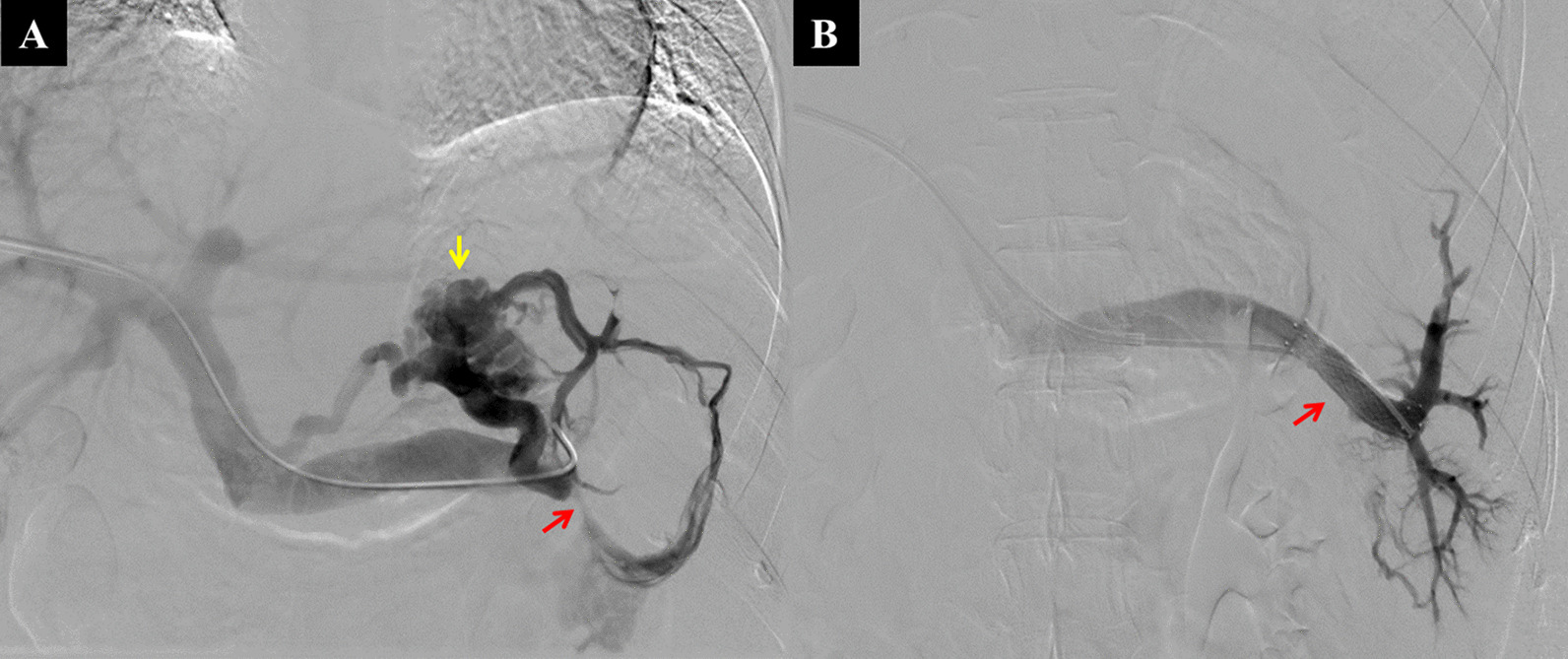
Percutaneous splenic vein stent implantation. A 36-year-old woman underwent percutaneous splenic vein stent insertion. **A** After fluoroscopy-guided selective intubation of the splenic hilum using a guide wire, splenic venography showed severe splenic vein stenosis (red arrow), short gastric veins, and varicose gastric fundal veins (yellow arrow). **B** After the splenic vein stent (red arrow) was inserted, the angiography showed that the splenic vein was unobstructed, and the varicose veins had shrunk significantly

After the patient was given local anesthesia with 2% lidocaine, the right portal vein branch was percutaneously punctured under the guidance of ultrasound or CT, and a 6F sheath was inserted. Portal vein venography was performed using a cobra catheter and the main portal vein pressure was measured. To observe the splenic vein patency and evaluate the varicose veins, fluoroscopy-guided selective splenic vein catheterization was performed at the splenic hilum to allow for splenic venography (Fig. 2). The splenic vein pressure was measured. If the splenic vein was severely stenosed (> 70%), a vascular stent (WALLSTENT™ Endoprosthesis, Boston Scientific, MA, Massachusetts, United States) was implanted to improve the splenic vein flow. Then, the splenic vein pressure measurement was repeated. The catheter sheath was withdrawn and coils were used to block the puncture tract of the right intrahepatic portal vein branch. Rivaroxaban anticoagulant therapy was administered from the second day postoperatively for 1 year.

### Splenectomy group treatment approach

After general anesthesia was administered, an L-shaped incision was made in the upper abdomen to explore the spleen and splenic blood vessels. The splenic artery was sutured and the ligaments around the spleen were cut off to prolapse it. Thereafter, the splenic and renal ligaments were cut and the splenic tissues were successively ligated to remove the spleen. A drainage tube was placed in the splenic fossa. Postoperatively, symptomatic treatment was provided, such as antibiotic prophylaxis, anti-infection measures, and pancreatin inhibition for 1 week.

### Conservative group treatment approach

Patients in this group were administered with proton pump inhibitors to inhibit gastric acid production, somatostatin to inhibit pancreatic enzyme secretion, gabexate to inhibit pancreatic enzyme activity, and antibiotics for infection prevention. Symptomatic treatments were provided concurrently, such as nutritional supplements, etc. Propranolol was required for primary prevention in patients who did not undergo surgery and who were at risk of gastrointestinal bleeding by gastroscopy. So in the conservative group, we recommended oral propranolol to reduce portal pressure and thus reduced the risk of bleeding.

### Evaluation

All patients followed up at the outpatient clinic. The follow-up time was determined based on the duration between the patient’s last medical history record and the outpatient follow-up visit. It also included the clinical manifestations, imaging findings, and biochemical examination reports that were related to PPH. We recorded the preoperative and postoperative (1 M, 3 M, 6 M) platelet count of patients in stent group and splenectomy group. We also recorded the spleen size of patients in stent group before and after surgery (1 M, 3 M, 6 M). Among them, the size of the spleen was measured on the coronal plane in CT images to measure the long diameter of the spleen (L: the maximum distance from the upper edge of the spleen to the lower edge). The thickness of the spleen was measured at the cross-sectional hilum level (T: the minimum distance from the spleen hilum to the outer edge of the spleen).With ongoing follow-up visits until January 2022, all the stent group patients were re-examined by endoscopic ultrasound and contrast-enhanced abdominal CT to assess the esophageal fundus and splenic veins at 1, 3, and 6 months postoperatively.

### Statistical analysis

The statistical software package SPSS version 17.0 (IBM Corp., Armonk, NY, United States) was used to analyze the data. The data measured from the normal distribution were expressed as x ± s, and the group t-test was used. The Chi-square or two-tailed Fisher's exact tests were used to compare rates. A P-value < 0.05 was considered statistically significant.

## Results

The operation was successful in all 21 patients included in the stent and splenectomy groups. Of the 9 patients in the stent group, the preoperative main portal vein pressures were all within the normal range, with an average of 16.5 ± 2.7 cmH_2_O. It was suggested that none of these patients had sinus or retrosinus portal hypertension. All 9 patients’ intraoperative splenic venography showed severe stenosis and/or occlusion of the main splenic vein (Fig. 2A). The splenic vein flowed to the portal vein through the short gastric vein and gastric varices. The intravascular splenic vein pressure in the splenic hilum was higher than normal, with an average of 33.03 ± 9.36 cmH_2_O, indicating that the portal hypertension was regional. Each patient had 1–2 self-expanding stents implanted that were 8–12 mm in diameter and 37–80 mm in length (average length, 62.78 ± 12.86 mm). After the stents were placed, splenic venography showed apparent vessel patency and the gastric varices had either shrunk significantly or disappeared (Fig. 2B). The average splenic hilar pressure was 20.66 ± 8.90 cmH2O, which was significantly lower than the preoperative pressure (P = 0.002, < 0.05).

During the follow-up period, none of the 9 patients had gastrointestinal bleeding recurrence. Re-examination through contrast-enhanced abdominal CT showed that the splenic vein stent patency rate was 100%, and the stents showed good positioning with no displacement or deformation (Fig. 1F–H, 1K-1M). Re-examination through endoscopic ultrasonography showed that all the varicose veins at the base of the stomach had shrunk by varying degrees, and the red color signs (RCS) regressed (Fig. 1I, J, N, O).The average platelet count at 1, 3, 6-months postoperatively were all significantly higher than the preoperative value(P < 0.05). However, compared with the stent group, the postoperative platelet count in the splenectomy group was significantly increased (P = 0.000, P < 0.05). The comparison of platelet count before and 1, 3, and 6 -months after procedures were shown in Table 2. The spleen volume did not change much in the stent group after surgery (P > 0.05). The changes of spleen volume in stent group were shown in Table 3.

**Table 2:** Changes of platelet count

	Stent group (*10^9/L)	P value*	Splenectomy group (*10^9/L)	P value*
Preoperative	88.11 ± 26.52		127.08 ± 46.24	
1 month after procedure	114.0 ± 33.21	0.014	423.50 ± 182.69	0.000
3 months after procedure	110.11 ± 30.23	0.021	464.17 ± 148.89	0.000
6 months after procedure	111.86 ± 28.12	0.038	445.0 ± 100.08	0.000

P value*: comparison of platelet count before and 1, 3, and 6 months after procedures

**Table 3:** Changes of spleen volume in stent group

	L (mm)	P value*	T (mm)	P value^#^
Preoperative	149.78 ± 20.29		44.0 ± 3.43	
1 month after procedure	149.89 ± 20.18	0.813	44.33 ± 3.97	0.563
3 months after procedure	146.67 ± 16.94	0.053	44.11 ± 3.37	0.855
6 months after procedure	144.29 ± 18.20	0.124	43.29 ± 4.27	0.873

P value*: comparison of the long diameter (L) of spleen before and 1, 3, and 6 months after procedures

P value^#^: comparison of the thickness (T) of spleen before and 1, 3, and 6 months after procedures

The complications of splenic vein stent placement mainly include: operation failure, splenic vein rupture, bleeding, infection, pancreatitis, stent displacement, stent restenosis, etc. However, these complications were not identified during the follow-up in the stent group During the follow-up period, the contrast-enhanced CT scans revealed that none of the patients had portal vein thrombosis or portal hypertensive gastropathy. The average length of postoperative hospital stay was 3.1 ± 1.4 days.

In the splenectomy group,none of them experienced gastrointestinal bleeding recurrence. Re-examination through endoscopic ultrasonography showed that the varicose veins at the base of the stomach had shrunk by varying degrees, and the RCS regressed too. Regarding the postoperative complications: 4 cases developed fever postoperatively (incidence rate, 33.3%); 1 case developed abdominal cavity infection, 1 case developed incision infection (postoperative infection rate, 16.7%); and 1 case experienced delayed abdominal hemorrhage, with approximately 2000 ml blood loss (incidence rate, 8.3%). To date, 2 patients had experienced portal vein thrombosis (incidence rate, 16.7%) during follow up. Following re-examination through endoscopic ultrasonography, none of the patients had portal hypertensive gastropathy. The average postoperative hospital stay duration was 16.1 ± 8.1 days, which was significantly longer than that in the stent group (P = 0.000, < 0.05).

In the conservative group, 1 patient underwent subtotal pancreatectomy and gastrectomy due to recurrent acute pancreatitis. During the follow-up period, endoscopic ultrasonography showed that 6 patients had mosaic-like changes to the gastric mucosa, indicating portal hypertensive gastropathy (incidence rate, 50.0%). 3 patients had symptoms of gastrointestinal bleeding: 1 case had upper gastrointestinal bleeding after 46 months of conservative treatment, mainly manifested by hematemesis and melena, with a bleeding volume of about 800 ml. Gastroscopy and CT examinations were performed at the local hospital, hemorrhage due to pancreatic portal hypertension was considered, and interventional embolization was given. Another case had upper gastrointestinal bleeding after 12 months of conservative treatment, mainly manifested by melena, with a bleeding volume of about 500 ml. After conservative symptoms such as medical hemostasis, the patient still had upper gastrointestinal bleeding repeatedly. At the 18th month, the patient underwent splenic artery embolization and splenectomy, and the patient did not have gastrointestinal bleeding symptoms ever since. The third case developed upper gastrointestinal bleeding after 72 months of conservative treatment, mainly manifested as hematemesis and melena, with a bleeding volume of about 1000 ml. Ultrasound gastroscopy showed multiple varicose veins in the fundus and body of the stomach, considering the bleeding caused by pancreatic portal hypertension. Symptomatic hemostasis was performed, and splenic vein stenting was performed after bleeding was controlled.

## Discussion

PPH is a type of regional portal hypertension. It is a disorder of the portal vein dominated by blood reflux from the splenic vein that is caused by pancreatic disease, and results in increased portal system pressure. Ru et al. [8] reported the prevalence of PPH was 2.7% in chronic pancreatitis patients. The characteristic clinical manifestations of PPH are the presence of primary pancreatic disease [9, 10], gastric varices, splenomegaly, and normal liver function. Related gastrointestinal bleeding occurred in 19.1% patients [8]. Although PPH is clinically rare, if upper gastrointestinal bleeding occurs, the prognosis is extremely poor [11–13]. In our study, 6 patients in the stent group had a history of gastrointestinal bleeding (incidence rate was 66.67%). They have a long-term chronic pancreatitis disease basis, and come to our hospital for splenic vein stent treatment when they have symptoms of upper gastrointestinal bleeding. Similarly, in the conservative treatment group, there were 3 patients (incidence rate was 25%) who had upper gastrointestinal bleeding during the conservative treatment process.

As patients with splenic vein occlusion have normal portal pressure and normal hepatic function, portal systemic shunting is not indicated [14]. Rozenblit et al. [15] reported a case of splenic venous hypertension had recurrent massive gastric variceal hemorrhage after treated by intrahepatic portosystemic shunting. Current clinical treatment methods for PPH include splenectomy, splenic artery embolization (SAE), endoscopic sclerotherapy, endoscopic ligation, etc. [16–18]. In clinics, splenectomy combined with pericardial vascular interruption is currently used as a treatment. When the spleen is removed, the systemic circulation pressure disappears, as do the splenic collateral circulation and retraction pressure. The portal vein pressure also decreases. However, as a surgical intervention, splenectomy is traumatic, and the postoperative hospital stay duration is prolonged. Furthermore, there are several postoperative complications, including bleeding, infection, pancreatic fistula, portal thrombosis, liver failure, etc. Our study also showed that there is a risk of bleeding and infection with splenectomy, and it may lead to portal vein thrombosis. Takahiro Sato et al. [14] reported that SAE is an attractive alternative treatment for gastric varices associated with splenic vein occlusion in patients at high surgical risk. Wu et al. [19]compared the outcomes of SAE and splenic vein stenting (SVS). Rebleeding was significantly less common in the SVS group. In our study, no rebleeding occurred in both stent group and splenectomy group. So splenic vein stent implantation and splenectomy may be both good ways to prevent gastrointestinal rebleeding.

More than 90% of PPH cases are caused by acute or chronic pancreatitis. Reports have shown that as long as the primary pancreatic disease is controlled, the PPH can be improved to a certain extent. However, in this study, 12 patients who were diagnosed with chronic pancreatitis were treated conservatively, and their PPH did not improve significantly after receiving treatment. Among these patients, 6 eventually developed portal hypertensive gastropathy, 3 occurred upper gastrointestinal bleeding, and 1 patient underwent subtotal pancreatectomy and gastrectomy due to disease progression.

Regional portal hypertension caused by different etiologies has different prognosis, which may cause gastrointestinal bleeding. Mizuno et al. [20] compared 536 post-pancreaticoduodenectomy patients and found that patients with splenic vein resection were more likely to develop varicose veins, bleeding, and thrombocytopenia than patients with splenic vein preservation. The regional portal hypertension caused by splenic vein stenosis with chronic pancreatitis is due to the obstruction of splenic vein. If the stenotic splenic vein can be recanalized, it will be possible to restore the blood flow to the liver in the splenic vein, thereby eliminating the cause of left portal hypertension, which is the starting point of our research.

Pancreatic edema and fibrosis can be caused by inflammation, cysts, and other diseases of the pancreas, thereby compressing the adjacent splenic vein. Inflammation may corrode the splenic vein wall, causing vessel damage and narrowing of the lumen. This causes secondary thrombosis and splenic vein lumen obstruction, resulting in obstructed splenic venous return, passive opening of the splenic vein branches, and, ultimately, regional portal hypertension [21]. Therefore, as long as the splenic venous return obstruction is relieved, the splenic vein pressure can be reduced, which effectively treats the regional portal hypertension. In this study, in 9 patients from the stent group, the blood flow from the splenic vein to the liver recovered well after the splenic vein stent was inserted, and the regional portal hypertension was significantly relieved. In addition, the average platelet counts increased.

Luo et al. [22] reported transjugular endovascular recanalization of splenic vein as a therapeutic option. In our study, CT- or ultrasound-guided percutaneous right portal vein puncture is also a safe and feasible procedure. Since there is no need to use TIPS technique, our method may simpler than the transjugular route. When there is severe splenic vein stenosis caused by chronic inflammation, passing the guide wire through the stenosis can prove difficult. Our experience with this procedure provided the following points: (1) Guide catheter use: with the support of the guide catheter, it is easier for the stent conveyor to enter the main splenic vein. (2) Repeated twisting of the super-smooth guide wire might be helpful. If the stenotic vessels still cannot be passed through, a 0.14-inch micro-guide wire with a micro-catheter can be used. (3) To ensure long-term stent patency, the patient can be treated with long-term anticoagulation for 1 year postoperatively. There is a controversy on anticoagulation therapy after portal vein stent placement [23]. In this study, after the stents were inserted, the gastric varices resolved and the gastrointestinal bleeding risk reduced significantly; thus, it was safe for anticoagulation therapy to be administered.

When the splenectomy and conservative treatment groups were compared, the stent group had the following advantages: (1) All the patients successfully underwent splenic vein stent insertion without any surgery-related complications, such as bleeding and infection. While the incidences of bleeding and infection in the splenectomy group were 8.3% and 16.7%, respectively. None of the patients in this group had secondary portal vein thrombosis, while 16.7% of the patients in the splenectomy group had splenic vein thrombosis, which may have aggravated the portal hypertension in the long term. (2) The stent group had an excellent outcome. During the follow-up period, all gastric varices were shrunk by varying degrees and the red color signs regressed, which were as good as splenectomy group and better than conservative group. During the follow-up, patients in the stent group did not reappear gastrointestinal bleeding after stent implantation. (3) The stent group had a shorter hospital stay duration and lower total treatment costs. In this study, the average postoperative hospital stay duration in the stent group was 3.1 ± 1.4 days, which was significantly shorter than that for the splenectomy group. There was no need to visit the hospital multiple times for subsequent treatment, and this would result in a significant reduction in the total medical expenses.

Compared with the stent group, the postoperative platelet count in the splenectomy group was significantly increased. Moreover, the spleen volume did not change much in the stent group after procedure. In addition, patients with splenic vein stent implantation have long-term risk of restenosis and need to use anticoagulants for a long time. Although restenosis and bleeding caused by anticoagulants had not yet occurred in patients in the stent group in this study, the shortcomings of these stent placements still require longer-term follow-up.

## Conclusion

The treatment of chronic pancreatitis with PPH through percutaneous splenic vein stent implantation has a high safety. It reduces the risk of gastrointestinal bleeding with minimal invasive. Moreover, it has a good therapeutic effect and quick recovery from less trauma, which saves hospitalization days and treatment costs. It is worthy of further clinical promotion. However, due to the small number of patients included in this study, a follow-up study with a large sample size is required to further demonstrate its long-term efficacy.

## Supplementary Information

**Additional file 1**. Changes of platelet count and spleen volume of each patient.

**Additional file 2**. General information of each patient.

## Data Availability

The datasets used in this study are uploaded as Additional file 1 and Additional file 2.
